# Comparison of the Effects of Remimazolam and Sevoflurane on Intraoperative Hemodynamics in Patients Undergoing Off-Pump Coronary Artery Bypass Surgery: A Retrospective Observational Study

**DOI:** 10.7759/cureus.82338

**Published:** 2025-04-15

**Authors:** Atsushi Miyazaki, Mai Hokka, Satoshi Mizobuchi

**Affiliations:** 1 Department of Anaesthesiology, Kobe University Hospital, Kobe, JPN; 2 Department of Anesthesiology, Kobe University Hospital, Kobe, JPN

**Keywords:** cardiac anesthesia, intraoperative hemodynamics, off-pump cabg, remimazolam, sevoflurane

## Abstract

Purpose

This study was conducted to compare the efficacy and safety of remimazolam with those of sevoflurane in patients undergoing off-pump coronary artery bypass (OPCAB) surgery.

Methods

This study was a single-center, retrospective observational study. Adult patients who underwent OPCAB surgery between April 2023 and July 2024 and received sevoflurane or remimazolam for maintenance of general anesthesia were included. The primary outcome was intraoperative average blood pressure (ABP). Secondary outcomes were amounts of norepinephrine and other vasopressors used during surgery, intraoperative heart rate (HR), fluid balance, central venous O_2_ saturation (S_CV_O_2_), score of Intensive Care Delirium Screening Checklist (ICD-SC) during the intensive care unit (ICU) stay, occurrence of postoperative acute kidney injury (AKI) and duration of ICU stay. All postoperative complications during the hospitalization period were evaluated.

Results

Thirty-six patients were included. Eighteen patients were administered remimazolam and 18 patients were administered sevoflurane for maintenance of general anesthesia. Intraoperative ABP was significantly higher in the remimazolam group than in the sevoflurane group (73±4 mmHg vs 69±6 mmHg, *P*=0.015). The amount of intraoperative norepinephrine was significantly smaller in the remimazolam group than in the sevoflurane group (910±638 μg vs 2041±927 μg,* P*<0.001). There were no differences in other outcomes and incidences of postoperative complications.

Conclusion

In OPCAB surgery, patients who received remimazolam for maintenance of general anesthesia achieved significantly higher average blood pressure than those who received sevoflurane, even though the amount of intraoperative norepinephrine was significantly smaller in the patients who received remimazolam than in the patients who received sevoflurane.

## Introduction

Remimazolam is a newly developed short-acting benzodiazepine anesthetic [1,2]. It was first approved in China in 2019 for sedation during endoscopy, and subsequently approved for general anesthesia in Japan in 2020, and its indication is expanding worldwide [3,4]. This drug has been reported to be superior to other anesthetics in terms of efficacy and safety, with the advantage of minimal effects on circulation and the presence of an antagonist [5,6]. Recently, there have been several reports on the use of remimazolam in anesthetic management of cardiac surgery, and remimazolam has been shown to be useful for circulatory stability and reduction of postoperative complications [7,8]. With regard to off-pump coronary artery bypass (OPCAB) surgery, there has been a report that maintenance of intraoperative mean arterial pressure (MAP) is important for the prevention of postoperative acute kidney injury(AKI) and that intraoperative hypotension is associated with a longer hospital stay [9]. Intraoperative maintenance of circulation is a major challenge in OPCAB surgery, but remimazolam may stabilize circulation during surgery compared to conventional anesthetics. Although there have been case reports on the use of remimazolam for anesthetic management of OPCAB surgery [10], there are few reports on its usefulness and safety. The present study was carried out to compare the circulatory stability and safety of remimazolam with those of sevoflurane in patients undergoing OPCAB surgery.

## Materials and methods

Design

This study was a single-center, retrospective observational study. Ethical approval for the study was provided by the Kobe University Hospital Ethics Committee on 18 September 2023 (approval no. B240048). Trained researchers reviewed patient medical records and anesthesia records and entered them into a database. Since all of the patients who underwent general anesthesia in the researchers’ center gave consent to the use of their data in the research, no new consent forms were obtained for conducting this study. Instead, information was provided to patients using an opt-out.

Participants

The study included the following patients: 1) adult patients who underwent elective OPCAB surgery during April 1, 2023, and July 31, 2024; 2) patients who received sevoflurane or remimazolam for maintenance of general anesthesia, regardless of the type of anesthetic induction drug. Patients who underwent emergency surgery, minimally invasive coronary artery bypass surgery, or on-pump beating surgery, and patients who received extracorporeal membrane oxygenation during surgery were excluded.

Data collection

Researchers obtained patient information from patient medical records and anesthesia records. Information on the following patient characteristics was obtained: age, sex, weight, height, vital signs, American Society of Anesthesiologists-Physical Status (ASA-PS), European System for Cardiac Operative Risk Evaluation II (EURO score II), medical history, preoperative left ventricular ejection fraction (LVEF), and hematology data. Surgical information, including operation time, type of procedure, intraoperative vital signs, and amounts of drugs and transfusions, and all information regarding the postoperative course until discharge from the hospital, was also obtained.

Outcome of the study

The primary outcome of the present study was intraoperative MAP. Secondary outcomes were amounts of norepinephrine and other vasopressors (ephedrine and phenylephrine) used during surgery, intraoperative heart rate (HR), urine output, fluid balance, lactate level immediately after surgery, central venous O_2_ saturation (S_cv_O_2_), bispectral index (BIS), score of Intensive Care Delirium Screening Checklist (ICD-SC) during the intensive care unit (ICU) stay, occurrence of postoperative acute kidney injury (pAKI) and duration of ICU stay. All postoperative complications during the hospitalization period were recorded as a safety evaluation. In this report, surgical time refers to the period from the start of skin incision to the end of surgery. The pre- and post-induction periods are not included in the analysis.

Anesthetic method

All OPCAB surgeries were performed under general anesthesia. The choice of general anesthetic maintenance medication was left to the anesthesiologist in charge. Only remimazolam was used for anesthetic induction and maintenance of general anesthesia in the remimazolam group. In the sevoflurane group, remimazolam, midazolam or propofol was used for induction of anesthesia and sevoflurane was used for maintenance of general anesthesia. Remimazolam and sevoflurane were titrated to achieve a BIS value of around 40-60. In both groups, the general anesthetic maintenance drug was switched to propofol before the end of surgery and the patient left the operating room under sedation. Opioids such as remifentanil and fentanyl were also used for general anesthesia induction and maintenance in both groups. The use of vasopressors was also left to the anesthesiologist, and blood pressure was generally maintained at around ±25% of the preoperative blood pressure.

Statistical analysis

All continuous variables are expressed as medians (IQRs) or means ± SDs. In comparisons between the two groups, statistical analysis was performed using the Mann-Whitney U test for continuous variables and the Chi-square test or Fisher's exact test for categorical variables. Analysis of covariance (ANCOVA) was used to compare the two groups for outcomes considered to be affected by operating time. MAP was calculated by averaging continuous minute-by-minute data of mean arterial pressure during the surgery. Mean arterial pressure was measured using an arterial line placed in the radial artery. ICD-SC scores were compared using the maximum score during the ICU stay. No statistical analysis for sample size determination was performed in this study, and a P value <0.05 was considered to indicate a statistically significant difference. Statistical analysis was performed using SigmaPlot 14.5 (SYSTAT software, CA, United States).

## Results

Thirty-six patients met the inclusion criteria. Eighteen patients were administered remimazolam (remimazolam group) and 18 patients were administered sevoflurane (sevoflurane group) for maintenance of general anesthesia. Table 1 shows a comparison of the demographic data in the two groups. There were no differences in patient characteristics between the two groups, including preoperative mean arterial pressure and cardiac function. The operation time was longer in the sevoflurane group than in the remimazolam group (402±56 min vs 347±60 min, P=0.007), but other factors were not significantly different between the two groups. The average intraoperative remimazolam infusion rate was 0.59±0.16 mg/kg/h, and the average sevoflurane administration concentration was 1.2±0.2%. 

**Table 1:** The demographic data in the two groups All continuous variables are expressed as medians (IQRs) or means ± SDs. In comparisons between the two groups, statistical analysis was performed using the Mann-Whitney U test for continuous variables and the chi-square test or Fisher's exact test for categorical variables. BMI: body mass index, ASA-PS: American Society of Anesthesiologists physical status, Euro SCORE II: European System for Cardiac Operative Risk Evaluation II, HD: hemodialysis, EF: ejection fraction, eGFR: estimated glomerular filtration rate, MAP: mean arterial pressure, HR: heart rate, IABP: intra-aortic balloon pumping, LAD: left anterior descending branch, LCX: left circumflex branch, RCA: right coronary artery.

	Remimazolam group (n=18)	Sevoflurane group (n=18)	P value
Patient characteristics			
Age (years)	72.5 (71-81)	73 (65-78)	0.10
Males, n (%)	15 (83.3)	16 (88.9)	1.00
BMI (kg/m^2^)	21.9±2.6	23.6±3.7	0.11
ASA-PS			
2, n (%)	4 (22.2)	4 (22.2)	1.00
3, n (%)	14 (77.8)	14 (77.8)	1.00
EuroSCORE II	2.40 (1.85-2.80)	1.30 (1.00-2.41)	0.07
Hypertension, n (%)	12 (66.7)	15 (83.3)	0.44
Diabetes mellitus, n (%)	8 (44.4)	9 (50.0)	0.74
Preoperative HD, n (%)	0 (0)	2 (11.1)	0.49
Preoperative EF, %	52.3±14.2	56.8±6.5	0.23
Preoperative eGFR, ml/ min/1.73m^2^	55.5±19.6	53.8±22.7	0.81
Preoperative MAP, mmHg	85±12	83±10	0.48
Preoperative HR, bpm	72±11	68±13	0.32
Operation data			
Operation time, min	347±60	402±56	0.007
Anesthetic induction drug			
Remimazolam (n, mg/kg)	18, 0.14	2, 0.12	-
Propofol (n, mg/kg)	0	11, 1.0	-
Midazolam ( n, mg/kg)	0	5, 3.0	-
Intra-operative drugs			-
Fentanyl (µg/kg)	13.3±5.5	11.1±3.3	0.16
Remifentanil (µg/kg)	58.5±23.1	48.5±10.6	0.10
Propofol (mg/kg)	2.2±1.3	3.2±1.9	0.09
IABP support, n (%)	0 (0)	1 (5.6)	1.00
Bypass artery			
LAD, n (%)	17 (94.4)	18 (100)	1.00
LCX, n (%)	17 (94.4)	17 (94.4)	1.00
RCA, n (%)	18 (100)	16 (88.9)	0.49

Outcomes

Intraoperative MAP was significantly higher in the remimazolam group than in the sevoflurane group (73±4 mmHg vs 69±6 mmHg, P=0.02). The results of analysis of covariance, in which the effect of the difference in operation times in the two groups was adjusted, showed that the adjusted MAP levels were 73 ± 1 mmHg in the remimazolam group and 69 ± 1 mmHg in the sevoflurane group (P = 0.03). Eighteen patients in the remimazolam group and 18 patients in the sevoflurane group received norepinephrine during the operation. The amount of intraoperative norepinephrine was significantly smaller in the remimazolam group than in the sevoflurane group (910±638 μg vs 2041±927 μg, P<0.001). Other outcomes were not significantly different between the two groups (Table 2).

**Table 2:** Results for primary outcome and secondary outcomes All continuous variables are expressed as medians (IQRs) or means ± SDs. In comparisons between the two groups, statistical analysis was performed using the Mann-Whitney U test for continuous variables and the chi-square test or Fisher's exact test for categorical variables. ANCOVA was used to compare the two groups for outcomes considered to be affected by operating time. MAP: mean arterial pressure, HR: heart rate, S_cv_O_2_: central venous oxygen saturation, BIS: bispectral index, ICD-SC: intensive care delirium screening checklist, pAKI: postoperative acute kidney injury, ICU: intensive care unit.

	Before adjusting for operating time			After adjusting for operating time		
	remimazolam group (n=18)	sevoflurane group (n=18)	P value	remimazolam group (n=18)	sevoflurane group (n=18)	P value
Primary outcome MAP(mmHg)	73±4	69±6	0.02	73±1	69±1	0.03
Secondary outcomes HR (bpm)	72±13	67±9	0.16	73±3	67±3	0.15
Norepinephrine (µg)	910±638	2041±927	< 0.001	798±192	1821±192	0.001
Ephedrine (mg)	9±8	11±11	0.97	9±3	11±2	0.67
Phenylephrine (mg)	1.0±1.3	0.4±0.4	0.88	1.0±0.3	0.4±0.3	0.10
Urinary output	495±469	569±453	0.63	495±469	569±452	0.27
Fluid balance (ml)	3087±774	3190±1009	0.72	3350±179	2927±179	0.12
Lactate level immediately after surgery	1.1±0.4	1.3±0.8	0.37			
S_cv_O_2 _(%)	72±9	71±8	0.74			
BIS	46±6	47±8	0.79			
ICD-SC	1 [0.75-2]	1 [1-2]	0.87			
pAKI (n, %)	3 (16.7)	2 (11.1)	1.00			
ICU stay (days)	4 [3-5.3]	4 [3-5]	0.53			

Safety evaluation

All postoperative complications are shown in Table 3. There was no significant difference between the two groups in the incidence of postoperative complications during the hospitalization period.

**Table 3:** Incidence of postoperative complications In comparisons between the two groups, statistical analysis was performed using the chi-square test or Fisher's exact test for categorical variables. pAf: paroxysmal atrial fibrillation, AV block: atrioventricular block.

Postoperative complications (n, %)	Remimazolam group (n=18)	Sevoflurane group (n=18)	P value
Graft dysfunction	3 (16.7)	1 (5.6)	0.60
pAf	1 (5.6)	4 (22.2)	0.34
AV block	1 (5.6)	0	1.00
Ventricular arrhythmia	1 (5.6)	0	1.00
Pancreatic enzyme elevation	1 (5.6)	0	1.00
Liver enzyme elevation	1 (5.6)	0	1.00
Aspiration pneumonia	0	1 (5.6)	1.00
Postoperative bleeding	0	1 (5.6)	1.00

## Discussion

The results revealed that intraoperative MAP in the remimazolam group was significantly higher than that in the sevoflurane group, and the amount of intraoperative norepinephrine was significantly smaller in the remimazolam group than that in the sevoflurane group. There were no significant differences between the two groups with regard to safety evaluation.

During OPCAB surgery, circulatory dynamics fluctuate greatly due to cardiac decompensation for graft anastomosis, and vasopressors, including norepinephrine, are frequently required [11,12]. One of the challenges of anesthetic management in OPCAB surgery is how to stabilize circulatory dynamics. In non-cardiac surgery, intraoperative hypotension has been shown to be associated with postoperative adverse events such as postoperative AKI [13,14]. With regard to OPCAB surgery, Xiao et al. found that maintenance of intraoperative MAP is important for the prevention of postoperative AKI and that intraoperative hypotension is associated with a longer hospital stay [9]. Therefore, stabilization of circulatory dynamics during OPCAB surgery is an important challenge for patient outcomes.

There have been several studies in which the effect of remimazolam and sevoflurane on hemodynamics during general anesthesia was compared. Ko et al. compared the effects of remimazolam and sevoflurane on hemodynamics during coil embolization of a cerebral aneurysm under general anesthesia [15]. The results showed that the use of remimazolam significantly reduced the incidence of intraoperative hypotension events. Miyoshi et al. compared hemodynamics during general anesthesia with remimazolam and conventional anesthetics in patients with severe aortic stenosis [16]. Patients in whom remimazolam was used for induction and maintenance of general anesthesia needed less intraoperative vasopressors than did those in whom propofol or sevoflurane was used. These studies indicated that more stable hemodynamics may be maintained during general anesthesia with remimazolam than during general anesthesia with sevoflurane.

On the other hand, there have been few reports about the use of remimazolam in OPCAB surgery. Oyoshi et al. reported two cases in which remimazolam was used to maintain general anesthesia in OPCAB surgery [10]. They reported that intraoperative hemodynamics, including hemodynamics during induction of anesthesia, were stable. Their report indicates that remimazolam enables the maintenance of excellent hemodynamic stability in patients undergoing OPCAB surgery. In the present study, the use of remimazolam enabled maintenance of high MAP and reduction in the amount of intraoperative norepinephrine in patients undergoing OPCAB surgery. Perioperative circulatory control may be difficult in patients with coronary artery disease due to systemic atherosclerosis, but, in the future, remimazolam may be useful for providing stable circulatory control in OPCAB surgery.

There are several limitations in the present study. First, since this study was a single-center, small sample size, retrospective study, the findings should be interpreted cautiously. Second, the difference in MAP between the two groups was only 4 mmHg, and it is necessary to verify whether this difference is a clinically meaningful value. Third, in the sevoflurane group, propofol and other drugs were used for induction of anesthesia, and the choice of these drugs may have affected the outcome. Fourth, the method of administering anesthetics, including remimazolam, is left to the anesthesiologist in charge, and administration methods are not standardized. Fifth, it is possible that the mean arterial pressure used in this study as an outcome does not accurately reflect intraoperative hemodynamic instability.

Although these limitations should be taken into account, it is meaningful that statistically significant differences were found despite the small sample size. To overcome these limitations, a sample size and a prospective design study will be needed to detect clinically meaningful blood pressure differences.

## Conclusions

This study compared the efficacy and safety of remimazolam with those of sevoflurane in patients undergoing off-pump coronary artery bypass (OPCAB) surgery. It was found that, in OPCAB surgery, patients who received remimazolam for maintenance of general anesthesia achieved significantly higher intraoperative mean arterial pressure than those who received sevoflurane and required less norepinephrine. The amount of intraoperative norepinephrine was significantly smaller in the remimazolam group than in the sevoflurane group. There were no differences in other outcomes and incidences of postoperative complications.
